# Dying with end stage kidney disease: factors associated with place of death on a palliative care program

**DOI:** 10.1590/2175-8239-JBN-2023-0015en

**Published:** 2023-10-20

**Authors:** Ana Cunha Rodrigues, Filipa David, Rita Guedes, Céu Rocha, Hugo M. Oliveira

**Affiliations:** 1 Centro Hospitalar Tondela Viseu, Portugal.; 2 Hospital Pedro Hispano, Matosinhos, Portugal.; 3 Hospital Pedro Hispano, Equipa de Cuidados Paliativos, Matosinhos, Portugal.

**Keywords:** Palliative Care, Kidney Failure, Chronic, Terminal Care, Conservative Treatment, Cuidados Paliativos, Falência Renal Crônica, Tratamento Conservador

## Abstract

**Introduction:**

End of life care of patients with end-stage kidney disease (ESKD) may be particularly challenging and requires the intervention of a specialized palliative care team (PCT).

**Objective:**

To characterize the population of ESKD patients referred to a PCT and evaluate the determinants of planned dying at home.

**Methods:**

We performed a retrospective observational cohort study of all patients with ESKD referred to our PCT between January 2014 and December 2021 (n = 60) and further characterized those with previously known ESKD regarding place of death (n = 53).

**Results:**

The majority of the patients were female and the median age was 84 years. Half of the patients were on conservative treatment, 43% were on chronic hemodialysis, and the remainder underwent hemodialysis on a trial basis and were subsequently suspended. Of those with previously known ESKD, 18% died at home and neither gender, age, cognition, performance status, comorbidities, CKD etiology, or treatment modality were associated with place of death. Anuria was significantly associated with dying at the hospital as was shorter time from dialysis suspension and death. Although not reaching statistical significance, we found a tendency towards a longer duration of palliative care follow-up in those dying at home.

**Conclusion:**

Dying at home is possible in a palliative domiciliary program regardless of age, gender, etiology of CKD, major comorbidities, and treatment modality. Anuria and shorter survival from RRT withdrawal may be limiting factors for planned dying at home. A longer follow-up by palliative care may favor dying at home.

## Resumo

**Introdução:**

Os cuidados de fim de vida em doentescom doença renal terminal (DRT) podem ser desafiantes e necessitar do apoio de uma equipa especializada em cuidados paliativos (ECP).

**Objetivo:**

Caracterizar a população de doentes com DRT encaminhada à ECP e avaliar os determinantes para um fim de vida planeado no domicílio.

**Métodos:**

Realizámos um estudo de coorte observacional retrospectivo dos doentes com DRT encaminhados à ECP entre janeiro/2014 e dezembro/2021 (n = 60) e caracterizámos aqueles com DRT previamente conhecida relativamente ao local de fim de vida (n = 53).

**Resultados:**

A maioria dos pacientes eram mulheres comidade mediana de 84 anos. Metade dos doentes encontrava-se em tratamento conservador, 43% em hemodiálise crónica e os restantes suspenderam diálise iniciada agudamente. Daqueles com DRT previamente conhecida, 18% morreram em casa. Não foi objetivada associação entre género, idade, cognição, status funcional, comorbilidades, etiologia da DRC ou modalidade de tratamento da DRT e o local de óbito. A anúria e a menor sobrevida após suspensão de diálise associaram-se a um fim de vida no hospital e verificámos uma tendência para o fim de vida em casa nos doentes com mais tempo de acompanhamento pela ECP.

**Conclusão:**

O fim de vida no domicílio é possível num programa domiciliário de cuidados paliativos, independentemente de idade, sexo, etiologia da DRC, principais comorbilidades e modalidade de tratamento. A anúria e o menor tempo de sobrevida após suspensão da TRS podem ser fatores limitantes. Um acompanhamento mais longo em cuidados paliativos pode favorecer o fim de vida no domicílio.

## Introduction

Chronic kidney disease (CKD) is characterized by its progressive nature. Although the trajectory of the illness is variable between patients, most are expected to reach end-stage kidney disease (ESKD) and need renal replacement therapy (RRT). Nevertheless, we are frequently confronted with frail patients reaching ESKD who may not benefit from these invasive treatments^1^ and with those on RRT and low quality of life who may benefit from its withdrawal. In both cases, supportive care must be offered.

In 2013, KDIGO (Kidney Disease: Improving Global Outcomes) Controversies Conference on Supportive Care defined its fundamental domains^2^. It was stressed that nephrologists have an important role in identifying candidate patients for a conservative approach, discuss their treatment options, address and control their symptoms and, when needed, recognize complex situations that should be managed by a specialized palliative care team (PCT). End of life care planing for those on conservative treatment or discontinuing RRT may be particularly challenging, with complex symptoms to address. Moreover, evidence suggests that many patients with ESKD would prefer to die at home than in the hospital, which may add to the complexity of end of life care in such patients^3^. In the present study, we aimed to characterize the population of ESKD patients referred to our PCT and evaluate the determinants for planned dying at home.

## Methods

We performed a retrospective cohort study of all patients with ESKD referred to the PCT of Unidade Local de Saúde de Matosinhos between January 2014 and December 2021 (n = 60) and further characterized those with previously known ESKD followed by the nephrology department regarding the place of death (n = 53). Data was obtained after a careful review of the electronic charts on relevant demographic and clinical variables.

The statistical analysis was performed using IBM SPSS^®^ version 25. Categorical variables are presented as frequencies or percentages and continuous variables are presented as means and standard deviation (SD) or medians and interquartile range (IQR) for normal and non-normal distributions, respectively. The chi-square test was used to evaluate associations between categorical variables and the Student’s t test or the Mann-Whitney test were used for continuous and categorical variables, depending on whether the distribution of continuous variables was normal or not. Statistical differences were considered significant when p < 0.05.

## Results

During the period under review, 60 patients with ESKD were referred to our PCT. The majority were female (55%), had a median age of 84 years (IQR 77-89), had preserved cognition (52%), and were partially dependent for usual daily activities (52%). The vast majority had previous nephrology follow-up (92%) and the most frequent CKD etiologies were diabetic nephropathy (30%), unknown (18%), and chronic interstitial nephritis (17%). Half of the patients were on conservative treatment, 43% were on chronic hemodialysis, and the remaining had hemodialysis on a trial basis and later discontinued. The majority also had other significant comorbidities besides ESKD such as heart failure, diabetes mellitus, previous cerebrovascular or coronary artery diseases, dementia, active neoplasia, or chronic pulmonary disease.

We further characterized ESKD patients with pervious nephrology follow-up regarding place of dead (n = 53) and found that only a minority of them died at home (21%). In our cohort, neither gender, age, cognition, comorbidities, CKD etiology, or treatment modality were associated with place of death (Table 1). Anuria was significantly associated with dying in the hospital (OR 8.2, p < 0.05) as was shorter time from dialysis suspension to death (median 5 days for those dying in the hospital vs 32 days for those dying at home, p < 0.05) (Table 1). Although not reaching statistical significance, we found a tendency towards a longer duration of palliative care follow-up for those dying at home (median follow-up of 52 days vs 18 days for those dying in the hospital, p = 0.06).

**Table 1:** Characterization of the ESKD patients regarding place of death

Characterization of the ESKD patients regarding place of death (n = 53)			
	Hospital (n = 42)	Home (n = 11)	p
**Age (years)**	83.5 (74-89)	85.3 (±3.8)	0.312
**Female**, *n (%)*	23 (55%)	4 (36%)	0.277
**Preserved cognition**, *n (%)*	21 (50%)	6 (55%)	0.788
**Comorbidities**, *n (%)*			
Heart failure	26 (62%)	4 (36%)	0.177
Diabetes mellitus	20 (47.6%)	6 (55%)	0.682
Cerebrovascular disease	15 (24%)	5 (45%)	0.728
Dementia	14 (33%)	4 (36%)	1
Coronary artery disease	16 (38%)	2 (18%)	0.296
Active neoplasia	13 (31%)	1 (9%)	0.251
Chronic pulmonary disease	11 (26%)	3 (27%)	1
**CKD etiology**, *n (%)*			
Diabetic nephropathy	13 (31%)	4 (36%)	0.730
Chronic interstitial nephritis	6 (14%)	2 (18%)	0.665
Unknown	5 (12%)	3 (27%)	0.34
Cardiorenal syndrome	3 (7%)	0	1
Hypertensive nephroangiosclerosis	3 (7%)	1 (9%)	1
Chronic pyelonephritis	2 (5%)	0	1
AD-PCKD	2 (5%)	0	1
ANCA-associated vasculitis	1 (2%)	1 (9%)	0.375
**Anuria (<100 mL/24h)**, *n (%)*	17 (40%)	0	0.01*
**Treatment modality**, *n (%)*			
Conservative treatment	20 (48%)	7(64%)	0.344
Chronic hemodialysis withdrawal	22 (52%)	4(36%)	
**HD vintage at suspension (months)**	48 (16.5-109.5)	4 (1-55)	0.088
**Time from HD suspension to death (days)**	5 (1.5-13)	31.5 (20-293)	0.011*
**Time from CT option to death (days)**	145 (80-716)	331 (±335.6)	0.685
**PCT follow-up (days)**	18 (2-117)	52 (23-276)	0.061

AD-PCKD: autosomal dominant polycystic kidney disease; CKD: chronic kidney disease; CT: conservative treatment; HD: hemodialysis; PCT: palliative care team. *p < 0.05.

## Discussion

Palliative care focuses on the prevention and relief of suffering and aims to improve the quality of life of patients, their families, and caregivers. Although historically applied to oncologic patients unsuitable for curative procedures, the role of palliative care has been increasingly recognized in the management of end-stage organ failure, such as ESKD^4^.

In CKD, palliative care can be used throughout the continuum of the illness^5^. From symptom management at all stages of disease to end-of-life care in ESKD patients on supportive treatment or withdrawing from RRT, the success of the intervention relies on collaboration between the nephrologist (with “primary palliative care skills”) and a palliative care specialist (for more complex situations)^6,7^. In fact, the literature suggests that the majority of patients with advanced CKD wish to be informed about their treatment options including dialysis withdrawal, seek end-of-life planning, and want their physical symptoms addressed by the nephrology team^3,8^. While the best time for engaging in such discussions remains to be determined, advance care on end-of-life planning seems to be important in selected ESKD patients. Sentinel events (such as multiple hospitalizations or acute illnesses) and a negative answer to the surprise question *(“Would you be surprised if this patient died within the next year?”*) may present and opportunity^3^. Although it has been shown that PCT consultation improves advance care planning, continuity of care of ESKD patients by a primary physician (usually their nephrologist) seems to reduce end-of-life care expenditures and invasive interventions^9^ - when end-of-life planning is documented and known to the medical team, adherence seems to be the norm^10^. Thus, it is reasonable to state that nephrologists should receive adequate training in advance care planning to improve care^11^.

Regarding the place of death, the majority of patients with ESKD would prefer to die at home or in the hospice rather than in the hospital^3^. In a study of ESKD patients who died in hospital yards, the most common symptoms reported were pain, agitation, dyspnea, nausea, vomiting, and pruritus^12^. Although these symptoms can be managed at home in many cases, they are sometimes difficult to control outside of hospital facilities. In our experience, a planned end-of-life at home requires a strong domiciliary palliative care program along with a motivated family or caregiver.

In our study, only a minority of the ESKD patients died at home (less than the 35% to 52% previously estimated to have this wish^8,13^). Contrarily to previous investigations where RRT patients were more likely to die in the hospital than patients managed conservatively^14^, in our cohort neither gender, age, cognition, comorbidities, CKD etiology, nor treatment modality were associated with place of death. On the contrary, anuria was associated with 8.2 higher odds of dying in the hospital. This observation is not surprising since anuria at the time of referral to the PCT anticipates more complex symptoms and a faster evolution to death from electrolyte imbalances, making it harder to plan for transition to residence. Similarly, a shorter time from dialysis suspension to death was also associated with dying in the hospital. Although not reaching statistical significance, shorter dialysis vintage at suspension and longer duration of palliative care follow-up seemed to favor dying at home. In our opinion, these observations underline, at least in part, the need for an adequate time to control major symptoms and prepare the residence to receive the patient before discharge. They may also reflect an overall less complex population dying at home, with greater residual kidney function (none of such patients were anuric, although residual kidney function was not measured).

Despite its strengths, the present study has several limitations. First, we were limited to data that were already collected. This hindered the analysis of individual preferences regarding place of death, usually not stated in clinical charts. As we believe that meeting individual preferences is more important than the actual place of death, this may be an important area for future research. Second, the small number of patients included and the absence of patients on peritoneal dialysis may affect the generalization of findings. Lastly, although we have a well-established palliative care service with a strong domiciliary program, this may not be the case in many other institutions, limiting the applicability of our conclusions.

To the best of our knowledge, this is the first work published in the literature on the determinants of planned dying at home in ESKD patients, and we showed that it can be done. We believe that this exploratory investigation can stimulate future investigations on end-of-life care in ESKD patients and expose the need for advance care planning for such patients. We hope that this is a first step in that direction.

## Conclusions

End of life care in ESKD is a small but important part of kidney supportive care. With the present study, we showed that dying at home in a palliative domiciliary program is possible regardless of age, gender, etiology of CKD, major comorbidities, and treatment modality. Anuria and shorter survival from RRT withdrawal may be limiting factors for planned dying at home. On the contrary, a longer follow-up by PCT may favor dying at home.
